# Factors contributing to vaccine hesitancy and reduced vaccine confidence in rural underserved populations

**DOI:** 10.1057/s41599-022-01439-3

**Published:** 2022-11-24

**Authors:** Renee Robinson, Elaine Nguyen, Melanie Wright, John Holmes, Catherine Oliphant, Kevin Cleveland, Mary A. Nies

**Affiliations:** 1 College of Pharmacy, Idaho State University, Anchorage, Meridian, and Pocatello, AK, ID USA; 2 College of Pharmacy, University of Alaska/Idaho State University, Anchorage, AK USA; 3 College of Health, School of Nursing, Idaho State University, Anchorage, AK USA

**Keywords:** Science, technology and society, Education

## Abstract

Vaccination remains one of the most effective ways to limit the spread of infectious diseases, and reduce mortality and morbidity in rural areas. Waning public confidence in vaccines, especially the COVID-19 vaccine, remains a cause for concern. A number of individuals in the US and worldwide remain complacent, choosing not to be vaccinated and/or delay COVID-19 vaccination, resulting in suboptimal herd immunity. The primary goal of this study is to identify modifiable factors contributing to COVID-19 vaccine hesitancy among vaccine-eligible individuals with access to vaccines in two under-resourced rural states, Alaska and Idaho. This qualitative study used semi-structured interviews with providers and focus groups with community participants in Alaska and Idaho. A moderator’s guide was used to facilitate interviews and focus groups conducted and recorded using Zoom and transcribed verbatim. Thematic, qualitative analysis was conducted using QDA Miner. Themes and subthemes that emerged were labeled, categorized, and compared to previously described determinants of general vaccine hesitancy: established contextual, individual and/or social influences, vaccine and vaccination-specific concerns. Themes (*n* = 9) and sub-themes (*n* = 51) identified during the qualitative analysis highlighted a factor’s contributing to COVID-19 vaccine hesitancy and poor vaccine uptake. Relevant influenceable factors were grouped into three main categories: confidence, complacency, and convenience. Vaccines are effective public health interventions to promote health and prevent diseases in rural areas. Practical solutions to engage healthcare providers, researchers, vaccine advocates, vaccine manufacturers, and other partners in local communities are needed to increase public trust in immunization systems to achieve community immunity.

## Introduction

Pandemics such as the 1918–1919 influenza, severe acute respiratory syndrome (SARS), H5N1 influenza (bird flu), and most recently, severe acute respiratory syndrome coronavirus 2 (SARS-CoV-2), the virus responsible for coronavirus disease 2019 (COVID-19), have been responsible for over 20-million deaths worldwide (Trimble, 2019; Lee et al., 2020; WHO, 2014; Wright et al., 2019; Wallerstein, 2017). Vaccination remains one of the most effective ways to limit the spread of infectious diseases, reduce mortality, and morbidity. Ongoing research suggests that 70% of the United States (US) population will need to receive the COVID-19 vaccine to achieve herd immunity; however, the projected COVID-19 vaccine uptake is far lower than 70% necessary (Reese et al., 2018; Godin and Kok, 1996).

Despite current vaccine availability, waning public confidence in vaccines, especially the COVID-19 vaccine, remains a cause for concern for rural communities. Individuals in the US and worldwide may choose not to be vaccinated, especially given the expedited vaccine development timeline and technologies employed (Fine et al., 2011). Vaccine hesitancy, defined as the delay or refusal to receive a vaccine, despite access and availability, is complex and context-specific, remaining one of the top 10 threats to global health (Trimble, 2019; WHO, 2014). To help identify and address vaccine hesitancy and improve vaccine confidence, a World Health Organization (WHO) Strategic Advisory Group of Experts (SAGE) workgroup was created and many of the primary factors contributing to general vaccine hesitancy were identified; however, additional factors appear to be contributing the COVID-19 vaccine hesitancy (WHO, 2014).

Research has shown that health decisions, such as whether to receive or not receive a COVID-19 vaccine, are highly influenced by a number of social and cultural factors (e.g., political ideology, past experiences with health services, family histories, the moral dilemma between individual autonomy and the greater public health) and that additional factors may be contributing to the increased patient hesitancy noted by healthcare providers (CDC, 2021; Karafillakis and Larson, 2018). The goal of “VAccination Challenges through Community-Identified NEeds (VACCINE)”, an investigator-initiated vaccine hesitancy study, funded through the Merck Investigators Studies Program Grant, is to identify, understand, and rectify modifiable barriers and factors contributing to COVID-19 vaccine hesitancy among vaccine eligible individuals with access to the vaccine. The primary goal of this paper is to identify and understand factors contributing to vaccine hesitancy in two rural states, Idaho and Alaska.

This study was undertaken both in person and via Zoom (an electronic meeting room) with individuals from southcentral Alaska and southern Idaho. Health equity concerns of individuals in both Alaska and Idaho are similar to those found nationally in other primarily rural, under-resourced communities (Lee et al., 2020). Within the context of the current study, members of the VACCINE research team (licensed pharmacists and/or nurses with significant public health experience) are part of the community of interest (healthcare providers and residents practicing in southcentral Alaska and southern Idaho), familiar with the needs of patients hesitant to receive the COVID-19 vaccine. Project team members have conducted similar qualitative studies in the past, have worked directly with these communities to provide immunizations, and understand the healthcare access, resource disparities, and community-level challenges that exist. The target research population, also part of the community of interest, includes (1) community members and healthcare providers eligible to receive the vaccine, with access to the COVID-19 vaccine and (2) healthcare providers (physicians, physician assistants, nurse practitioners, nurses, and pharmacists working primarily in the community setting) caring for individuals eligible to receive the COVID-19 vaccine, who have access to the COVID-19 vaccine, but who have not yet received the COVID-19 vaccine.

## Methods

This study employed two qualitative methods, used semi-structured interviews with healthcare providers and focus groups with community participants in two under-resourced states, Alaska and Idaho. All focus groups and semi-structured interviews were conducted by core research team members, Renee Robinson (pharmacist) and Mary Nies (nurse), faculty at Idaho State University who has worked with and for this and similar under-resourced communities (e.g., Indigenous communities and rural residents). Both individuals are sensitive to and understand research participant challenges (reflexivity), trained to appropriately frame the research problem, and collect data in a culturally sensitive, respectful manner.

To ensure consistency in the data collection process, a moderator’s guide was used to facilitate interviews and focus groups conducted via Zoom in 2021. Interviews were recorded and transcribed verbatim. Thematic, qualitative analysis was conducted using QDA Miner. The themes that emerged were labeled and categorized into areas reviewed and confirmed by members of the research team. IRB was obtained and consent was obtained from all interview participants.

### Ethical approval

This study was granted expedited approval with a waiver of written consent by the Idaho State University Investigational Review Board (IRB) and is subject to established University research governance procedures. Participants and/or their legal guardians consented to participation at the time of interview and/or focus group scheduling (verbally) and verbal consent was confirmed and documented again prior to the interview and/or focus group initiation. All research was performed in accordance with relevant guidelines/regulations applicable to human subject participation (e.g., the Declaration of Helsinki).

### Theoretical framework

Community-based participatory research (CBPR), a well-developed model of relational research, offers a valuable framework for conducting research with community members to elicit understanding and identify stakeholder preferences and concerns. Utilization of the proposed qualitative methods within a CBPR framework will help identify the unmet needs of rural community members in the context of lived experience (Wright et al., 2019; Wallerstein, 2017; Reese et al., 2018). The World Health Organization (WHO) Strategic Advisory Group of Experts (SAGE) framework for vaccine hesitancy served as the strongest driver for the proposed research and assessment including contextual influences; individual and/or social group influences; vaccine and vaccination-specific issues. Two interpersonal behavioral theories, the Health Belief Model (HBM) and the Theory of Planned Behavior (TPB), were used to guide the interpretation of individual behavioral variation (Godin and Kok, 1996). We focused on the HBM and TPM factors impacting readiness to act: perceived susceptibility, perceived severity, perceived benefits, cue to action, and self-efficacy (Costa, 2020; Rosenstock et al., 1988). Through the TPB lens, we focused on the individual’s attitudes, subjective norms, and individuals perceived behavioral control. Lastly, the Consolidated Framework for Implementation Research (Damschroder et al., 2015), was used to ensure that the proposed intervention strategies address cultural, environmental, and other pertinent factors contributing to vaccine hesitancy.

### Advisory Board

Thirteen healthcare providers and/or community leaders engaged in the community and/or statewide COVID-19 vaccination efforts were recruited to the VACCINE Advisory Board. Advisory Board members were convened on three separate occasions, two times for this project, to guide the development of the interview and focus group moderator’s guide, and once to assist with the interpretation of qualitative data to guide the development of the Vaccine Hesitancy Survey. The final Advisory Board included two physicians, two pharmacists, an epidemiologist, State Immunization Task Force leads, the State Commission on Aging, the Director of the State Commission on Aging, and State Directors of the American Association of Retired Persons.

### Participant selection

A judgment or purposive sampling approach was used to identify adults (18 years of age and greater) with access to available COVID-19 vaccine (i.e., Moderna, Pfizer, or Johnson and Johnson) at the time of the study (government subsidized and/or insurance covered vaccine) from both urban and rural communities in Idaho and/or Alaska to assess access barriers COVID-19 potential differences in perceived susceptibility, severity, benefits, and risk. Informational flyers about the study were posted and shared by providers in the four-partner healthcare facilities and were shared on social media (Facebook and Google) by the University Marketing Department. Interested individuals then contacted the investigators, were screened for eligibility, and scheduled for one of the six focus groups for community members or seven interviews for healthcare providers.

Seven healthcare providers, two physicians, one mid-level provider, two pharmacists, two nurses, and one community health worker from five different healthcare facilities (tribal healthcare system, federally qualified healthcare center, privately funded healthcare facility, chain pharmacy, and a quality improvement organization) across Alaska and Idaho currently providing COVID-19 vaccinations were invited to participate in clinical service-focused interviews regarding COVID-19 vaccine hesitancy and delivery.

### Data collection

Co-developed moderator guides, piloted in one focus group and one interview, were used by the two primary VACCINE Qualitative Research team members (Drs. Robinson and Nies) to conduct the seven 45-min semi-structured provider interviews and six 90-min virtual patient focus groups using Zoom, a collaborative cloud-based, videoconferencing service offering a secure, recordable, online meeting platform, over a 7-week period in Spring 2021. Prior to the interview, verbal consent was obtained from participants and the study focus was discussed. During the interview, providers were asked: How have you been personally impacted by COVID-19 (health, economics, return to normality)? Who do you trust the *most* for COVID-risk & COVID-vaccine information? Who do you trust the *least* for COVID-risk & COVID-vaccine Information? Who are the *key people/sources* that influence your decision to recommend a COVID-19 vaccine? Note that no repeat interviews were completed and that transcripts were not returned to the participants for comment; however, audio recordings and field notes were assessed to ensure accurate information capture.

During the focus group, community members were asked: How have you been personally impacted by COVID-19 (health, economics, return to normality, cues to action)? Do you plan to get/receive the COVID-19 vaccination (perceived susceptibility)? What is the number one reason behind your decision to get the vaccination (individual’s attitudes, perceived benefits, condition severity)? Or not to get the vaccination? Who do you trust the *most* for COVID-risk & COVID-vaccine information? Who do you trust the *least* for COVID-risk & COVID-vaccine Information? Who are the *key people/sources* that influence your decision to get a COVID-19 vaccine (self-efficacy, subjective norms, and individual’s perceived behavioral control)? How does the government influence your decision to obtain a COVID vaccine? Have reports you heard or read in the newspaper or media prevented you from getting a vaccine (cues to action)?

The number of participants is based on prior work with the goal of achieving data and theory saturation as no new codes or themes were identified in the data, data repetition was noted, and identified codes and themes were exemplified in the data. Zoom recordings were subsequently uploaded to Rev, a cloud-based transcription service, and data was transcribed verbatim with over 99% accuracy. Patient identifiers were removed from interview transcripts, files were uploaded and stored in a secure, password-protected cloud-based site and subsequently uploaded directly into QDA Miner.

### Data analysis

A thematic analysis protocol was used to analyze data. Two coders systematically generated descriptive and analytic themes using a three-stage process: (1) line-by-line coding of the text for meaning and context, (2) development of descriptive themes and creation of code grouping based on differences, similarities, established literature (SAGE, TPB, and HBM), and (3) finally analytic themes are generated, identifying abstract messages and themes. Patterns and dominant concepts that emerged during analysis, and resultant themes and sub-themes were then compared to the established HBM, TPB, and SAGE theoretical frameworks focused on vaccine hesitancy: contextual influences (perceived susceptibility, perceived severity, perceived benefits), individual and/or social group influences (self-efficacy individual’s attitudes, subjective norms, and individuals perceived behavioral control), and vaccine and vaccination-specific issues.

## Results

Twenty-seven individuals, 52% women, ranging from 18 to 80 years of age, participated in one of the six-virtual focus groups conducted over a 2-week period. Seven healthcare providers involved in vaccine administration and currently working within a healthcare facility, 86% of women, ranging from 25 to 66 years of age, participated in a semi-structured virtual interview conducted over a 4-week period. All participants were actively involved in the administration of the vaccine, counseling of patients, and were aware of barriers to vaccine delivery within their clinical setting and community. Interviews lasted between 22 and 70 min (average 47 min) and focus groups lasted between 46 and 97 min (average 73 min). The result, nine-identified themes, and 51-sub-themes categorized are labeled and presented in Table 1. Data and theory saturation were attained, as no new codes or themes were identified in the data, data repetition was noted, and identified codes and themes were exemplified in the data. Approximately 62% of the main themes were related to interview questions, and the remaining 38% of themes emerged during the analysis of interviewees’ responses. The themes and sub-themes identified during the qualitative analysis highlighted a number of factors contributing to COVID-19 vaccine hesitancy and poor vaccine uptake, including modifiable and non-modifiable barriers. Relevant influenceable and/or modifiable barriers were grouped into three main categories: confidence, complacency, and convenience.

1. Vaccine confidence commonly refers to the trust patients, families, and providers have in recommended vaccines, and the processes and policies that led to vaccine development, manufacturing, approval, and administration. Under vaccine confidence, three main themes emerged: trust, information and resource needs (self-efficacy, individual’s attitudes), and fear of vaccine safety and side effects (individuals perceived control). A number of contextually based trust concerns were identified by participants relating to group influences, information sharing, vaccine, and vaccination-specific issues, for example:
  “I don’t know what the answer is as far as regaining trust with the CDC or the WHO, or Fauci, or just any level of government at this point, because it was so politicized from the beginning”.
  “I think the main concern for me is not knowing what information to trust…”—33-year-old childbearing female
  “My husband and I have had to regroup and then just make the decisions that are best for us and our immediate home and nuclear family. I don’t rely on social media for my information. I don’t trust the algorithms.”—50-year-old female
  A number of information needs were identified by participants including agenda-based, biased, and filtered information, highlighting the need for clear, unbiased, trustable sources of information that demonstrates long-term safety and efficacy, for example:
  “[People come to pharmacists for] free advice. I mean, you don’t have to make an appointment. You can call it, you can walk up, you can do whatever you want. And so, especially at the retail level, [patients] really will trust whatever you say.’—Pharmacist
  “I’ve been getting information through my parents and then also through my in-laws, that’s where, I know my mother-in-law, she has daughters with disabilities so she does a lot of research about vaccines. I don’t know where my dad gets his information…. But I do trust my mother-in-law in terms of how she looks into each ingredient she kind of reads about… it does seem like she does put a lot of work into studying it.”—22-year-old female
  “I think self-report data is fine and it’s helpful, but I think I would prefer to see a longitudinal study where a follow-up is a full physical to check all vitals and to see if there are any issues or concerns and could those be related to the reception of the vaccine? I mean, because that’s what they do in some longitudinal studies is follow-up like that when it comes to health.”—48-year- old female
  “Not enough time has elapsed to know what the long-term effects of having had COVID are, to know how long your natural immunity lasts, to know how all the COVID vaccines work, or to know if there’s side effects. I mean, so I think it’s time. It’s not necessarily more information or access to information or whatever, it’s just we can’t speed that up.”—33-year-old female
  “The biggest scare for all of them is, uh, they’ve just heard bad things about people having bad signs and the thrombocytopenia and, and, and, you know, the reaction is the biggest scare and trying to reassure them that it has been hard because you can show them the data, you can explain to them, but then reach that we’re here to watch you”—Medical Provider
  We have a lot of people say, well, they skipped a phase. And what I’ve tried to explain to him is that the phase they skipped is the one that usually they do to make sure is this worth it for us or not.”—Nurse
2. Complacency is the low perceived risk and/or worry about getting COVID-19 virus disease and the perceived efficacy of treatment. Under the category of complacency two-main themes emerged: the importance of individual-level choice for health-related decisions (perceived susceptibility, severity, and benefits), the role of big companies and government in individual-level health decisions, and the perceived need for the COVID-19 vaccine.
  A number of contextually based concerns related to [individual] choice were identified by participants including concerns that the information being provided by industry, governmental agencies, and vaccine and vaccination-specific concerns was biased and that the messaging is coercive, for example:
  “I’ve been coerced by a few different individuals in my life, my kids and my doctor, to the point where, “Oh yeah, it’s your choice, but you really should get this.” Now a little bit more by my kids who are both in the medical field who have both had their vaccinations. They won’t be around me because I haven’t been vaccinated. Well, that doesn’t make any sense to me because if they’re vaccinated, they should be fine. Why would they be concerned about me?”—64-year- old male
  “I try to find my own information independently. There’s some weight to Dr. Fauci and what he recommends. But I try to lean more on my own primary care doctor and my own health situation in evaluating the risks of getting COVID, or in my case getting COVID again, versus getting the vaccine and how necessary that is.”—32- year-old male
  “I don’t care if you’re 85 years old and you deserve to live the rest of your life until you naturally pass away or get hit by a car or however God has it planned out for you. So I just, I wonder if we’ve gone too far…”—85-year-old-female
  “My family members who are at high risk have already gotten vaccinated. So that puts me at ease of, okay, they got a little bit more protection from the coronavirus. So that influences me to be a little more relaxed or I don’t feel pressured to go get vaccinated.” —36-year-old female
  “Probably only adults who can legally make their own decisions who want it. I don’t think any minors who can’t make their own choices yet, can’t consent to things, I don’t think they should get it, but only adults and only people who want to.”—40-year-old female
  A number of contextually based concerns related to regulations were identified by participants suggesting group influences, information needs, and vaccine-specific issues, for example:
  “So my issue personally is that I don’t want to set the precedent for the government, that they can require us to do something to our bodies.”—32-year-old female
  “I don’t think that in order to go on vacation, I don’t think that you should have to show a vaccine card. I can understand vaccines have been mandatory for certain things. You can’t enroll your kid in school without certain vaccines. And, they’ve been mandatory for a while for certain things, but something so new… you can’t just expect everyone to just jump on board and trust something that’s only been around for a couple of months.”—43-year-old female
  “I can understand if this was more urgent here, why [regulations] would be a necessity, but I don’t feel like, especially in Idaho where we are, it seems a little over the top to have a mandate about it, but I don’t know.”—36-year-old male
  A number of contextually based concerns related to perceived vaccine efficacy and perceived vaccine need were identified by participants suggesting group influences, remaining information sharing, vaccine, and vaccination-specific issues, for example:
  “If I was over 65 or if I had diabetes, there’s plenty of people out there that are afraid of this, that have a reason to be afraid of this. And I think it’s great that we have a vaccine for those people, but I don’t think that it should be pushed on people that don’t feel they need it, just the flu. I don’t get the flu vaccine either”—33-year-old male
  “Do I trust big pharma? No…. I’m sure it’s [the vaccine] pretty darn safe and effective and all of that. So, yeah, I think in general there’s a lot of ethical issues with big pharma. But I don’t believe that they’re putting bad stuff in it [the vaccine]”—52-year-old male
  “If they were saying that this vaccine made you 100% not contagious and that it would stop this spread and it would save lives… but it’s saving lives of people that are highly susceptible to this and that are taking the vaccine to lower their risk of being hospitalized.”— 44-year-old female
3. Convenience: The quality of the service (real and/or perceived) and the degree to which vaccination services are delivered (e.g., appealing, affordable, convenient, and comfortable) the decision to vaccinate determines the priority that an individual person places on vaccination. Under the category of convenience, three main themes emerged: access, availability, and messaging. A number of contextually based concerns related to access were identified by participants suggesting group influences on vaccination and information, for example:

“I seek out resources that I trust, and it might be WebMD or Mayo Clinic or other national organizations, and then just periodically try to understand how vaccines work, how does this vaccine work, what are the risk factors. I seek out information as well instead of just whatever the newspapers or media serves up to me.”—52-year-old female

“Sometimes, you know, you end up with more people than you anticipate, and you need to pull that extra supply of band-aids, gauze, alcohol wipes, gloves, uh, wipes, uh, hand sanitizer, things that are just naturally in a clinic. It’s not uncommon to get somewhere and be like… the clinic froze up this weekend. So guess what? We’re moving over to the tribal hall…”—Nurse

“I’ve even had some people that are just so frustrated with the whole trying to get an appointment has been really frustrating for people. And I’ve had some people say, well, by the time I get an appointment, I’ll be dead. So, I guess I shouldn’t worry about it. You know, like some elderly people have just made some really frustrating, you know, they’re frustrated. And so, you know, I always tell people and I have, this is a true story. I gave a shot to a 101-year old woman a few weeks ago.”—Healthcare Provider

**Table 1:** Themes and Sub-themes.

Theme	Sub-themes
COVID-19 Infection	Impact on person; Impact on community; Impact on Family; Guidelines
Vaccine History	History Against Vaccines; History Pro Vaccines
Trust	Trust Builders; Trust Breakers
Information	Information needs; Information access; Misconceptions; Information bias; Information transparency; Contradictions in information provided; Data Collection
Choice	Barriers to individual choice; Risk versus benefit; Timing; Influencers of Choice; Political influence on choice; Rule-making agencies influence on choice
Fear	Fear of vaccine safety; Fear of COVID-19 infection; Risk to others; Risk to self; Fear of outside parties having control
Resources	Media resources; Social media resources; Public health campaigns; Newspaper or television news; General internet search; Internet news source; Family, Friends, Healthcare providers, Government; Industry; Primary Literature, CDC and similar governmental websites
Impact of Vaccine on…	Travel; Job; Life; Livelihood; Sense of security
Concerns	Concerns with vaccine efficacy; Social stigma; Concerns regarding restrictions; Concerns associated with site of administration; Cost of vaccine administration; Cost of vaccination to taxpayer; Access to vaccine

A number of contextually based concerns related to availability were identified by participants suggesting group influences, information needs, and vaccine-specific issues, for example:

“I don’t know the word incentive means something more to me than just not having a barrier to it. I think incentive means, I get something else rather than just the vaccine with no cost, but I think I wouldn’t risk my health for that initiative, unless it was really, really good, probably it’d have to be more than what’s reasonable.”—47-year-old female

“At least in the beginning, they were only giving us the highest available options from testing centers… so that immediately skews the view that everybody has COVID…”—50-year-old female

Lastly, a number of contextually based concerns related to messaging were identified by participants suggesting group influences, information needs, and vaccine-specific issues, for example:

“I’ve got a 15-year-old son. He’s been absolutely indoctrinated by his school [to get the vaccine].”—50-year-old male

“I feel strange about there being [media] ads about getting the vaccine”—50-year-old female

“So I heard that somewhere that celebrities would come out and say, you need to take the vaccine, but they’re not telling you which one, nor have we seen the drug company itself come out and say, ours is the best or you need to take ours. Because my understanding is they can’t do that until it’s fully FDA approved, hopefully I’m wrong.”—46-year-old female

“Advertisements I’ve seen, because they’re not by those companies, they don’t mention side effects or anything like that”—65-year-old male

“They’re not getting a consistent message yet. And as such, if there’s any reluctance, they’re like, you know, you know, but I see, I have seen people who did not want it in December, come in January or February after they’ve seen people respond, you know, well, or, you know, that it didn’t kill us, that we’re all alive. I got, well, you know, that we’re all alive and that didn’t turn us into green monsters or anything. And we survived and we didn’t get COVID.”—Medical Provider

“So maybe like the messaging around what people end up having to pay for versus what’s covered, and then telling people that they need the vaccine, there might be a disconnect. And like, you’re telling me I need to get vaccinated, but you’re not covering all of them [family members on the same visit]. So why are there any other opportunities that you see as far as kind of maybe messaging or education or things that we could kind of, maybe [provide] better support, like for vaccines in general for vaccine hesitancy in general?”—Pharmacist

## Discussion

Factors such as individual vaccine confidence and complacency have been shown to impact the uptake of preventative care services such as immunizations and were of the greatest concern in our population. It is important to note that the fortuitous timing of the existing delivery system expedited delivery development and pressure to rapidly disseminate vaccines to local and state governments. However, a number of opportunities have been identified by patients and providers to increase confidence and reduce hesitancy.

### Confidence

Patients have increasingly expressed the desire to be more involved in their treatment decisions, especially in critical situations such as with COVID-19 risk mitigation strategies and related decisions (Kucukarslan et al., 2012). Medical decision-making is the ability of a patient to understand the benefits, risks, and treatment/intervention options (Society of Medical Decision Making, 2021). Information is necessary to make an informed medical decision; however, in the case of the pandemic and COVID-19 vaccine, healthcare providers and the community members are learning together, and individuals feel that they do not have the information necessary to make an informed decision, and are that their right to choose is being infringed upon (Rahman et al., 2021).

It is important to note that vaccines usually go through a long, over 10-year, rigorous approval stepwise process to assess safety and efficacy (FDA, 2019, 2022) In phase 1 (preclinical) safety of the vaccine is assessed, phase 2 efficacy is tested in a small group, phase 3 efficacy is tested in a larger group, and phase 4 (post-licensure) ongoing study of safety and efficacy after-market release is evaluated (FDA, 2019, 2022). Unlike previous vaccines, the currently approved COVID-19 vaccine by Pfizer and Moderna vaccine was developed in <12 months, with distribution and administration to healthcare providers beginning in December 2020 (CDC, 2021). In our study the distrust people have of drug manufacturers, the government, and the media have contributed to significant vaccine hesitancy and decreased vaccine confidence in the COVID-19 vaccine, especially by those in groups at lower risk of serious COVID-19-related health complications (middle-aged, young adults, and adolescents).

Our study concerns that information is not being openly shared with the public, that the information needed to make an informed healthcare decision is not provided, and that the information that is provided, especially by the media, is contradictory in nature; leaving individuals confused, fearing for the safety of their family and friends. Many steps within this process could be improved, information better disseminated and opportunities are taken to acknowledge why guidance has changed and what information and support people need to make an informed decision.

There are a number of opportunities to identify and address these risk factors including more transparent, consistent messaging and information, to better support patient medical decisions. Individuals need to feel comfortable with the risk they assume and to feel comfortable they have to have enough information to assess the risks. If this is a global condition, where is the global response? And how can we make sure information is being openly shared with everybody in hard-to-reach rural areas and that steps are taken to correct the misconceptions of patients, providers, and healthcare leaders, especially in rural areas? As a social currency, trust, and relationships evolve, and we found collaboration is just as important, contributing to improved health outcomes.

### Complacency

Though over 334 million doses of the COVID-19 vaccine have been administered in the US, only 159 million individuals are fully vaccinated (48.5% of the population). Low perceived COVID-19 risk and low perceived treatment efficacy, contribute to the sub-optimal vaccination rates across the country; lower those seen earlier in the pandemic (66%), lower than those seen with other vaccinations which range from 15% to 90%, and lower than rates necessary to establish herd immunity (70%) (Fine et al., 2011; Mayo Clinic, 2021; Statista, 2021; Our World Data, 2021).

In our study, perceived efficacy, choice, and regulations contributed to individual-level complacency. Most focus group participants felt they were at minimal risk either based on age, current health status, and/or community they lived in, believing that if infected the condition would be self-limiting and complications minimal. This low-perceived risk was also seen in healthcare providers in our study. This is consistent with what is seen in the literature, despite the increased risk to frontline healthcare providers and the current prioritized availability of the COVID-19 vaccines to healthcare providers, a number of healthcare providers have not been vaccinated, potentially putting themselves, their patients, and the healthcare systems at risk (Larson et al., 2015; Washington Post, 2020). Vaccine confidence among healthcare providers may be heightened through targeted discussion, identifying and addressing concerns, and involving healthcare providers more in vaccination decisions and patient-facing messaging (CDC, 2021; Karafillakis and Larson, 2018; Our World Data, 2021). Americans generally have a high level of trust for physicians and other healthcare providers more so than public authorities and the government (Larson et al., 2015; The Washington Post, 2020).

Complacent focus group participants reported they did not feel they had enough information to make truly informed, personalized healthcare decisions, unable to adequately weigh vaccine risks and benefits. Direct messaging to healthcare providers has also been shown to decrease vaccine hesitancy in patients; however, it is important to note that simply delivering factual information is inadequate and there is no guarantee that information is getting out to the rural areas. Vaccine confidence requires patient-provider discussion, the provider eliciting and addressing patient concerns such as what would happen if they were injured by the vaccine, and patient involvement in the decision-making process (Larson et al., 2015; Stefanoff et al., 2010).

### Convenience

In our study, convenience was related to access, availability, and messaging. This is consistent with want is reported in the lay and scientific literature that across all income levels access to vaccines and trusted public health information remains a barrier to COVID-19 vaccination (Deloitte Insights, 2022; Edge et al., 2017; Local Government Association, 2022), which suggests that a number of controllable factors influence consumer sentiment around vaccine uptake and confidence (Marzo et al., 2021; Al-Mohaithef and Padhi, 2020).

More and more patients are turning to pharmacists to receive immunizations across their lifespans (Mayo Clinic, 2021; Statista, 2021; Our World Data, 2021; Larson et al., 2015). New recommendations from the Advisory Committee on Immunization Practices (ACIP) highlight the abilities of pharmacists to assess, influence, and expand access to vaccines.

## Conclusion

Vaccines are an effective public health intervention to promote health and prevent diseases; however, for them to be taken they must be administered. To achieve community immunity, public trust in the immunization and immunization system is needed to address vaccine confidence issues raised by patients and improve immunization convenience to address complacency barriers. Practical solutions to increase vaccine confidence, identified directly by vaccine-hesitant individuals in our study and those in other vaccine hesitancy studies, have the potential to reduce hesitancy and improve vaccine uptake, especially with COVID-19 vaccination. Though the themes and sub-themes were as we expected, the weight placed on vaccine confidence and the related complacency (perceived susceptibility, severity, and benefits) by patients needs to be matched with interventions and related educational resources.

## Data Availability

Datasets generated during and/or analyzed during the current study are not publicly available due to small sample size, type of research, and potential identifiability but de-identified data sets will be made available from the corresponding author on reasonable request.
